# Breast cancer: a randomized controlled trial assessing the effect of a decision aid on mammography screening uptake: study protocol

**DOI:** 10.3389/fonc.2023.1128467

**Published:** 2023-04-24

**Authors:** Sandrine Hild, Delphine Teigné, Emilie Ferrat, Anne-Sophie Banaszuk, Karine Berquet, Aline Lebon, Emmanuelle Bataille, France Nanin, Aurélie Gaultier, Cédric Rat

**Affiliations:** ^1^ General Practice Department, Faculty of Médecine, Nantes, France; ^2^ Research Department, University Hospital of Nantes, Nantes, France; ^3^ Clinical Epidemiology ans Ageing (CEpiA), University Paris-Est Creteil, INSERM, IMRB, Paris, France; ^4^ Regional Organization in Charge of Cancer Screening Programmes, Angers, France; ^5^ Department of Statistics and Studies, Health Insurance System, Nantes, France; ^6^ Research Department, Methodology and Biostatistics Platform, University Hospital of Nantes, Nantes, France; ^7^ National Institute for Health and Medical Research/INSERM U1302 Team 2, CRCINA, Nantes, France

**Keywords:** breast cancer screening, organized screening, general practitioner, randomized controlled trial, shared medical decision making, decision aid

## Abstract

**Introduction:**

Breast cancer (BC) is the primary cancer among women. The World Health Organization recommends a bilateral screening mammogram every 2 years for women aged 50 to 74 years. However, it has been shown that there is an absence of information about the benefits and risks of screening. Shared medical decision-making is important to ensure patients are involved in the decision process. Decision aids can facilitative this decision-making process. This article presents a protocol to evaluate the effect of a decision aid on participation rates in the French organized BC screening program.

**Methods and analysis:**

Design and setting. The design is a 2 arm randomized controlled study, performed in the Pays de la Loire region (French West Coast). Randomization will be based on general medicine practices (Primary Care).

**Participants:**

Women aged between 50 and 74 years, eligible for BC screening. In this region, there are 75000 women, and 2800 general practitioners eligible for recruitment.

**Intervention:**

In the « Decision aid for organized cancer screening » arm, the intervention will distribute invitation letters to eligible women combined with the provision of decision aid to these women and their general practitioners and an incentive to implement shared medical decision-making. In the « Standard organized cancer screening » arm, only the screening invitation will be sent to eligible women.

**Primary endpoint:**

BC screening participation rates will be assessed after an 18-month follow-up period.

**Statistical analysis:**

In this non-inferiority trial, the percentage of women who are up-to-date with their screening at 18 months after the intervention will be compared across arms using a generalized mixed linear model.

**Discussion:**

The research team expect to demonstrate that providing a better explanation of the benefits and risks of BC screening is not at odds with screening participation. The study results should help policy makers thinking about implementing shared medical decision-making within the framework of organized BC screening programs in the future.

**Ethics and dissemination:**

On 6 December 2021, the protocol received a favorable opinion from the French Committee for the Protection of Persons (2021-A01583-38). This study is registered with ClinicalTrials.gov, number NCT05607849. (Version 1, November 7, 2022; https://www.clinicaltrials.gov/ct2/show/NCT05607849). The study findings will be used for publication in peer-reviewed scientific journals and presentations in scientific meetings.

## 1 Background

In 2020, breast cancer (BC) was the most commonly diagnosed cancer in women worldwide and was responsible for 1 in 6 cancer deaths (1). It was also the leading cancer in terms of incidence and mortality in French women (2).

International randomized trials have shown the effectiveness of BC screening using mammography, reporting a 15% to 20% decrease in specific mortality after 10 years of follow-up (3–8). To improve screening, the French authorities set up a national organized screening (OS) program in 2004 (9). The program targets women aged 50 to 74 years and follows World Health Organization (WHO) recommendations by inviting women at average risk for BC to have a bilateral mammogram every 2 years (10). It also provides reminders for women who have not spontaneously participated in screening within the recommended time intervals. All these invitations are sent by post. Current participation was 45.6% over the 2019-2020 period with a target of 65% (11).

Even within a framework for promoting screening, participation in screening remains an act of voluntary care and results from a health choice. In France, a citizen consultation highlighted dysfunctions in the current organization of screening and the subsequent consequences. These included a misunderstanding of the issues, and the absence of information about the benefits and risks of screening in the invitation letter sent every 2 years (12). The literature points out that the benefit/risk ratio of screening is difficult for a woman to predict from an individual perspective (3, 4, 13–16). Although participation in OS decreases mortality directly related to BC, it has significant disadvantages including false positive results, overdiagnosis and overtreatment which impact the patient’s quality of life (17). Health authorities recommend that these disadvantages are discussed in the invitation sent to these women, so that they have balanced and complete information, and can make an informed choice whether or not to participate in screening (12, 18).

Many authors have worked to improve information given to women and their involvement in the decision-making process. Shared decision-making is considered one of the fundamental approaches to improve health care quality (19, 20) Decision aids (DAs) can facilitate Shared Medical Decision-Making (SMDM). DAs should simply set out the best available evidence and the implications in terms of risks and benefits (21–23). Several DAs dealing with BC screening have been developed internationally (21, 23, 24). In 2018, the National Cancer Institute (INCa) funded the development of a DA specific to the French context as part of a call for research projects. The resulting DA was the “Discutons-mammo.fr” website, built in a collaborative and multi-professional way, and in accordance with international standards (25). The literature highlights that SMDM is best achieved if (i) women are informed about the existence of DAs and encouraged to discuss the OS with a healthcare professional (“coaching”) (26), (ii) health professionals are informed of the existence of DA, the different stages of the SMDM and the importance of their implementation (27). Finally, the use and dissemination of DAs requires evidence of their benefit, or at least evidence that DAs do not interfere with recommended care practices.

The primary objective of this study is to evaluate the impact sending a letter informing women of the existence of the DA “Discutons-mammo.fr” has on the BC screening participation rate in an organized setting, in routine practice and in the general population.

The secondary objectives are (i) to evaluate the predictive factors of using OS, and (ii) to measure the effectiveness of the intervention on SMDM implementation.

## 2 Methods and analysis

### 2.1 Design

The design is a non-inferiority, cluster, randomized controlled clinical trial with 2 parallel groups, performed in the Pays de la Loire region (West Coast of France) composed of 5 departments.

### 2.2 Setting

In France, all patients over the age of 16 must follow a coordinated care pathway. General practitioners (GP) are at the center of this system. They coordinate access to care and specialists, are involved in screening and must be consulted as a priority. The GP system is based on a reciprocal choice: that of the patient and that of the GP.

The study is based on the regular GP/patient pair to measure the effect of the intervention. A cluster is defined as a group of GPs within the same primary practice address.

### 2.3 Structure in charge of the organization of screening

OS management is entrusted to regional organizations in charge of cancer screening called CRCDC (“*Centre Régional de Coordination de Dépistage des Cancers*”). These organizations are in charge of collecting screening data for all women eligible for BC screening and follow-up cases with a positive or abnormal result. They collect information from health care providers (health insurance, pathology and cytology laboratories, hospital information systems, GPs, and cancer registries) involved in screening.

### 2.4 Participants

Women aged between 50 and 74 years at the start of the study, who are eligible for BC screening, live in the Pays de la Loire region, and are registered with a participating GP will be eligible for inclusion in the study. Any women who are being followed-up for BC or an anomaly, have a medical or family history of BC, have refused or whose GP has refused to participate in the study, have participated in other studies on BC screening, or are under guardianship or curatorship will not be eligible for inclusion. Participating GPs will be the registered GPs for the included women practicing in the Pays de la Loire region. The inclusion and exclusion criteria for women and GPs are shown in **Table 1**. The list of eligible women will be extracted from the CRCDC. The association regular GP-eligible women will be realized by the health insurance system.

**Table 1:** Inclusion and exclusion criteria for GPs and women.

	General practitioner	Women
Inclusion criteria	Specializing in General Medicine (excluding Special Practice Mode)	50 to 74 years old
	Working in Pays de la Loire	Residing in Pays de la Loire
	Having seen more than 100 different patients in the year prior to enrolment	Eligible for organized screening for the month in question
		Present on the patient list of a participating GP
		Affiliated to the health insurance system
Non-inclusion criteria	Refusing to participate in the study	Having a regular GP refusing to participate in the study
	Working in a Health Center	Affiliated with a health center
	Participating in other breast cancer screening studies	Participating in other breast cancer screening studies
		Under curatorship-tutorship
		Excluded from organized screening because they have a history of breast cancer
		Having ongoing follow-up (breast cancer or abnormality or medical surveillance or high-risk woman)

GP, General Practitioner.

### 2.5 Randomization

GPs will be clustered within practices to avoid contamination bias stemming from shared tracking mechanisms and communication among GPs within a practice. As a result, two GPs working in a given practice will be assigned to the same study arm. The women will then be allocated to the same arm as their GP.

### 2.6 Intervention and control procedures

One intervention arm and one control arm are planned.

In the “Decision aid for organized cancer screening” group (intervention arm), the intervention will combine sending letters to women and their GP. Women will receive their invitation to have a mammogram accompanied by a flyer mentioning the existence of the DA and encouraging them to consult their GP to make a shared decision. Physicians will receive a letter informing them of the correspondence their patients have received, the existence of the DA, a presentation of the different stages of SMDM and an incentive for their implementation.

In the “Standard organized cancer screening” group (control arm), the intervention will be limited to sending invitation letters, corresponding to the national standard (9).

### 2.7 Participant blinding and informed consent

**Figure 1:**
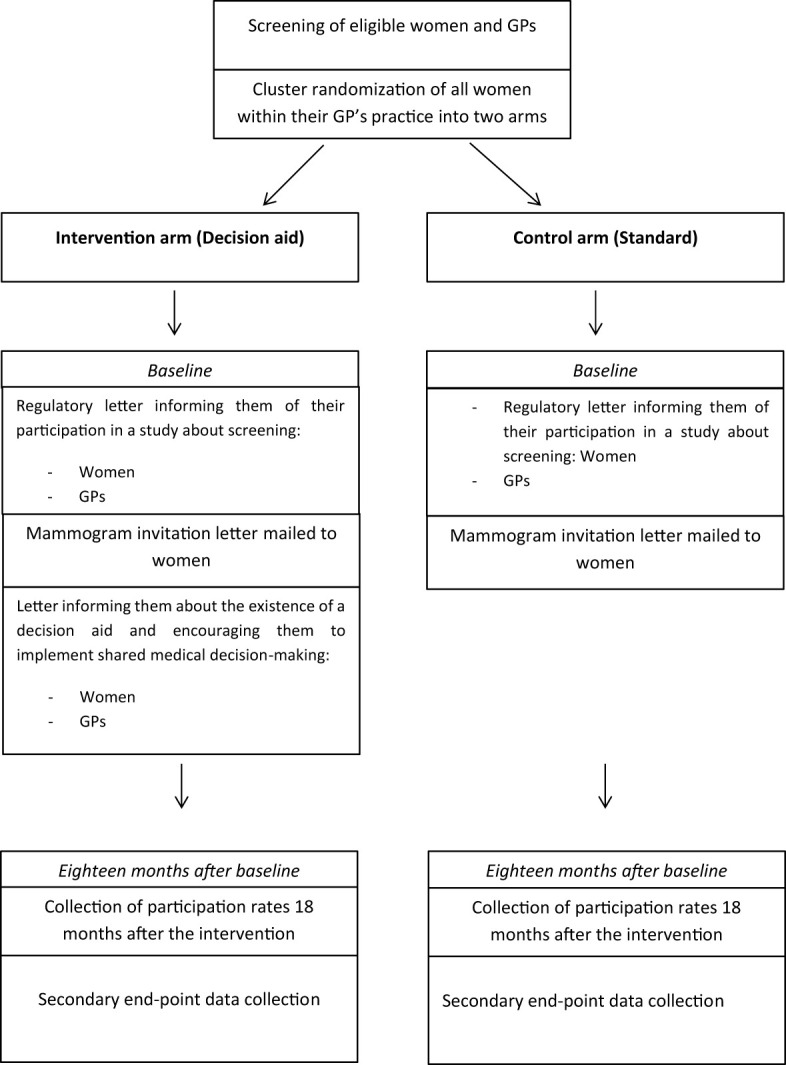
Study design.

The GPs and women in the intervention and control arms will receive a regulatory letter by post informing them that they are participating in a study on screening (**Figure 1**). Opposition to participation in the study is possible in both arms for women and GPs.

### 2.8 Objectives, primary endpoint, and judgement criterion

#### 2.8.1 Primary objective and primary endpoint

The main part of this study is to assess whether sending information to women and GPs mentioning the existence of DAs and encouraging SMDM implementation along with the screening information letter (intervention arm) affects participation rate in BC OS, compared to only sending the invitation letter to eligible women (control arm) (see **Supplementary Material 1**).

The primary outcome is the participation rate in BC OS, in each arm during the 18 months following the invitation being sent. This duration takes into account the waiting times observed to benefit from screening mammography (see **Supplementary Material 1**).

#### 2.8.2 Secondary objectives and evaluation criteria

##### 2.8.2.1 Assessing the predictive factors of the use of organized screening

The first secondary objective is to assess the predictive factors of OS use. Several studies have reported social inequalities in screening (28, 29). Suggested factors include increased age (30) and lower socio-economic status (31). Proxies of economic status are complementary health insurance status and French DEPrivation index of the place of residence. Therefore, as an associated secondary outcome, the study plans to collect the participation rate according to age and socio-economic level (beneficiaries of the Complementary Solidarity Health and municipality of residence according to the deprivation index).

In addition, the irregularity of women’s medical follow-up (32), the presence of a chronic pathology (33, 34) or psychiatric disorders (35, 36), and previous non-participation in screening (32) have also been reported as explanatory factors for non-participation in screening. Data will therefore also be collected over the 18-month period before and after the intervention to assess the health of participating women based on the Charlson Comorbidity Index (37) using the French health data collection system. Data will include: (i) the number of consultations and home visits to/by different healthcare professionals (GPs, midwives, gynecologists), and the number of journeys by taxi or medical transport, (ii) the prescription of certain reimbursed treatments and examinations performed (see **Supplementary Material 2**), (iii) participation in other cancer screenings.

##### 2.8.2.2 Measure SMDM implementation and assess the level of decision-making conflict

The second secondary objective is to measure the effect of the intervention on SMDM implementation and quality, as part of an ancillary study. While the use of DAs is still marginal in occupational practices, the study should provide a better understanding of how such a tool is used and perceived when it is made available to healthcare professionals.

To assess the SMDM quality, a composite criterion will be used. The validated Shared Decision Making Questionnaire (SDM-Q-9) (38) will be used to evaluate SMDM implementation. The validated Decisional Conflict Scale (DCS) will be used to assess decision-making conflict (39). Intent to participate will be measured using a multiple-choice question. Knowledge about OS and women’s doubts, questions and concerns will be assessed and collected using a questionnaire created for the study. Secondary outcomes will be SDM-Q9 and DCS scores, intention to participate and level of knowledge. This will be measured 2 months after the intervention, in a sample of women from each study arm (see **Supplementary Material 1**).

### 2.9 Collected data source

The CRCDC and the French Health Insurance System will collect the data needed to conduct the research in their databases. These data will be transferred to an open project space within the National Health Data System (NSDS) allowing their analysis. This data transfer will be carried out in accordance with the data protection regulations that govern personal data protection in the European Union. The Health Insurance data managers will pair the data collected from these databases at the patient level and proceed to data pseudonymization before analyses by the statistician.

### 2.10 Statistical analysis

All data will be described globally and by randomization arm. The research is a non-inferiority clinical trial. The statistical unit is the woman eligible for screening. The materiality threshold will be set at 5% in a bilateral situation. Statistical analyses will be performed using R 3.6.0 software (R Foundation for Statistical Computing, Vienna, Austria. URL https://www.R-project.org/).

#### 2.10.1 Main criterion

The percentage of women having undergone BC screening, 18 months after the intervention, will be compared between the arms using a generalized linear mixed model, to adjust the analysis for the random effect of the practice.

A two-sided 95% confidence interval of the difference in participation rates between the two arms will be calculated. The lower bound of this confidence interval will be compared to the non-inferiority margin. Non-inferiority will be demonstrated if the lower bound of the confidence interval is greater than the margin of non-inferiority defined at 2%, on the Intention to Treat (ITT) population and Per-Protocol population. If non-inferiority is demonstrated and if the participation rate in the intervention arm is higher than the participation rate in the control arm, the hypothesis will be verified with a superiority test (bilateral at 5%, according to the same model as that of the non-inferiority analysis) (40). If the test is significant, it will be concluded that the participation rate in the intervention arm is significantly higher than the participation rate in the control arm.

#### 2.10.2 Exploratory secondary analyses

Participation rates in exploratory subgroup analyses will be studied in the same manner as the primary endpoint. The same margin of non-inferiority will be used. There is no provision for alpha risk adjustment for these analyses. The different subgroups are: (i) women aged 50 to 59/60 to 69/70 to 74 years at baseline, (ii) beneficiaries/not beneficiaries of the Complementary Solidarity Health at baseline, (iii) women who consulted/did not consult at least one GP, gynecologist or midwife (during the follow-up period for women who did not have a bilateral mammogram during the 18 months, or from baseline until the date of the bilateral mammogram for women who had the screening) (iv) women residing in a municipality with a low deprivation index (per quintile of the deprivation index) (41).

Predictors of OS use will be assessed using a multivariate generalized mixed model fitted to the physician as a random effect, and as fixed effects: (i) the number of visits to the treating physician, GP, gynecologist, or midwife in the 18 months prior to enrolment, and from baseline to bilateral mammography or to the end of the follow-up period for women who did not have bilateral mammography, (ii) the woman’s age at baseline, (iii) the presence of a chronic condition measured *via* chronic drug use and testing, (iv) previous participation in OS.

A stepwise algorithm based on the Akaike information criterion (AIC) will be implemented to select variables that provide meaningful information independent of the main factors.

SMDM quality will be measured using the DCS and SDM-Q9 scales, responses to participation intent and knowledge of women about the OS. It will be compared between groups using a Student superiority test.

The care pathway of the women responding to the questionnaires will be studied and in particular the concordance between patients’ intention and effective participation in BC screening.

#### 2.10.3 Analysis population

Statistical analyses will be performed on all randomized physicians and patients included in the study. As data are collected on medico-administrative databases, very little missing data is expected (only the very rare case of women changing reimbursement schemes). If these women did not have a mammogram as part of the OS at the time of their change of plan affiliation, they will be considered non-participants for the assessment of the primary endpoint in the ITT analysis. Women who died during the study period and had not had a mammogram at the time of death, will also be considered non-participants for the assessment of the primary endpoint. The same applies to women who underwent individual screening during the study period.

A sensitivity analysis on a Per-Protocol population will be performed by removing from the analysis patients for whom a return mail has been received (for example “No longer living at the address indicated”)), patients who have moved outside the Pays de la Loire region, patients who have changed regular GP, patients whose regular GP has ceased to practice during the study period or has died, patients who have been individually screened, who do not enter OS for known medical reasons after the start of the study, or who died during the follow-up period.

### 2.11 Statistical justification for sample size

#### 2.11.1 Main study sample

Based on a participation rate in 2020 of 49% for the five participating departments (11) assuming a margin of non-inferiority of 2 points to demonstrate that participation is non-inferior in the intervention arm, and considering a power of 80% and a bilateral alpha risk of 5%, the number of women to be included is equal to 9,808 in each arm, being a total of 19,616 women.

There are 2,847 GPs in the five departments (42). Considering that GPs operate from 1,570 different practices (42) the number of women targeted per practice will be 48. With an average cluster size of 48 women and an intraclass coefficient of 5%, the number of subjects to be included to account for this cluster randomization must be multiplied by 3.35, or 65,647 women (32,824 per group).

The number of GPs included will be approximately 2,847 in the five departments. The recruitment potential is approximately 25,000 women per month (personal communication from the CRCDC). The inclusion will therefore be done over 3 consecutive months.

#### 2.11.2 Ancillary study sample

In her article, Pérez-Lacasta observed a difference in the decision-making conflict scale score between women who received information and those who did not of 18.53 vs. 13.77 (standard deviation at 20.25) (43). On these assumptions, 762 responses would be required to arrive at a significant difference.

The ancillary survey conducted by questionnaire to assess SMDM quality will be carried out on an independent sample of 5,000 women, equally divided between the 2 arms. Based on a response rate of 20%, the number of questionnaires collected will be 1,000.

### 2.12 Justification of the non-inferiority margin

In a non-inferiority trial, the margin chosen must meet two criteria: (i) be less than the differences observed in the superiority trials already conducted, and (ii) be less than the Minimal Clinically Important Difference.

International evidence on whether the use of DAs changes participation in the OS is scarce, without clear direction. It may therefore result in an increase (44) or a decrease in participation rates (45). Recent studies do not show a change in participation rates when women are invited to participate in decision-making (43, 46, 47).

The non-inferiority margin was set at 2% in agreement with the BC OS medical experts at the CRCDC. This is the largest difference in clinically acceptable OS participation. The Cochrane literature review reports an average of 1 death averted per 2,000 women screened over 10 years (44), the observation of 10 healthy women who would not have been diagnosed if they had not participated in screening and who will be treated unnecessarily (44). Thus, of the 75,000 women who will be included in the study, and according to our participation hypotheses, a 2% decrease in participation in the intervention group would imply the non-screening of 750 women. No additional deaths from BC are expected following the procedure, but between 3 and 4 women will not be treated unnecessarily.

The trial will therefore be conclusive if the lower bound of the two-sided 95% confidence interval of the difference in participation between the randomization arms is greater than 2.

### 2.13 Status and study schedule

The beginning of the intervention will correspond to letters being sent to the included women and their GPs (T0).

The ancillary study will begin 2 months after T0, with a follow-up at 2 months.

Patients will be followed for 18 months. The freezing of the OS participation database within 18 months will be carried out 30 months after the intervention (M30). This schedule takes into account the time required for data entry. All statistical analyses and valuations of results will be conducted from M30.

**Table 2** presents the time schedule for enrolment, intervention, and assessments.

**Table 2:** Time schedule for enrolment, intervention and assessments.

	Study period									
	Enrollment		allocation	Postallocation						Closeout
**TimePoint**		_t_1_	0	t_1_	t_2_	t_3_	t_4_	t_5_	… T_18_	T_30_
**Enrollment:**										
Eligibility screen		X								
Inform consent (main study) Inform consent (ancillary study)			X		X		X			
Allocation			X							
**Interventions:**										
Decision aid for organized cancer screening				X	X	X			…. X	
Standard organized cancer screening				X	X	X			…. X	
**Evaluations:**										
Proportion of women aged 50 to 74 who had a screening mammogram 18 months after the procedure.										X
Participation rate 18 months after the intervention according to age, socio-economic level, medical follow-up, presence of a chronic pathology, psychiatric pathology, previous participation in screening.										X
SDM-Q9 and DCS scores 2 months after the start of the intervention.										X
Answers to the intention to participate questionnaire, answers to the knowledge questionnaire 2 months after the start of the intervention.										X

SDM-Q9, Shared Decision Making Questionnaire; DCS, Decisional Conflict Scale. X = realization of the stage.

### 2.14 Patient involvement

Women were involved in designing and developing the DA.

### 2.15 Data storage and retention

Directly identifying and pseudonymized data relating to women and professionals willing to participate in the research will be kept in active database, in the CRCDC and French health insurance system information systems, up to two years after the last publication of the research results or, in the absence of publication, until the final research report has been signed. Pseudonymized data will also be kept in additional archives by archiving on paper and computer media for a period of fifteen years. These durations are based on the regulations applicable to research compliant with MR003 Reference Methodology, according to deliberation 2018-154 of May 3, 2018.

This study protocol has been prepared according to the 2013 SPIRIT guideline for clinical trial protocols (48). It was recorded in ClinicalTrials.gov (number NCT05607849, November 7, 2022; Version 1).

## 3 Discussion

### 3.1 Results and expected benefits of the DEDICACES study

The DEDICACES (“*DEcision partagée dans le cadre du DépIstage du CAnCEr du Sein en soins premiers*”) study should make it possible to evaluate whether the provision of a French DA (in accordance with international standards, built with patients and professionals) modifies participation in BC OS. At the end of the study, the research team expects to demonstrate that providing a better explanation of the benefits and risks of BC screening is not at odds with screening participation. Some women may choose not to participate, while others may choose to participate. In total, the average participation could therefore remain equivalent to the current participation rates. In addition, providing a DA without modifying participation would be significant progress in informing women and reducing decision-making conflict (and therefore better adherence). In this scenario, DA and SMDM on cancer screening for women would also meet societal demands for greater ethical practice in medical care for individuals.

While formalized SMDM practices in France are still rare, the study should make GPs aware of SMDM in BC screening. It will thus increase GP expertise on this topic and enable them to better answer their patients’ questions potentially leading to an increase in the number of women participating in screening.

Finally, the main perspective of the study is the generalization of DAs, which will be made available to women and healthcare professionals involved in BC screening.

Foreign literature has fed into the reflection of this study. International evidence on whether DA use modifies screening participation is scarce and results are contradictory (24, 44, 45, 49). A Cochrane literature review (44) reported that better informing women about their cancer risk and screening may increase participation, reporting a low level of evidence based on 3 studies (44, 50). On the other hand, the DECIDEO study born conducted in France reported a (limited) decrease in participation, estimated at 2% (45). Controlled trials have been performed evaluating tools to measure the effectiveness of shared decision interventions. These improve women’s knowledge about participation options and promote clear choices for BC screening (43, 51–53). On the other hand, the results for decision regret and decision conflict are more variable (43, 49, 52, 54, 55). There is uncertainty about whether interventions targeting both patients and healthcare professionals increase SMDM, compared to an intervention targeting either member of the pair (27). Furthermore, while factors associated with low participation have been widely described (28, 29, 56–58) literature does not report a relationship between DA availability and improved use of screening mammography for women meeting the criteria associated with lower participation.

Overall, these studies reveal the need for a robust and large-scale evaluation as we propose in this protocol. The central role of the regular GP in the care pathway, makes France an ideal location to conduct this study.

### 3.2 Strengths and limitations

The proposed research is characterized by the robustness of the design. Randomization is performed on GP medical practices with the aim of limiting confounding bias between arms. Research is also distinguished by its power; it is conducted in a region of western France, with differences in ease of access to healthcare professionals and screening participation. The trial will include several thousand women, about 4 to 30 times more than other studies evaluating DAs for screening mammography (24). Finally, the study is characterized by its reproducibility. The design is built with the intention of screening, and the intervention is easily reproducible.

There are some limitations. The content of the consultation with the GP cannot be studied. The ancillary study, which will be conducted on a small sample of women, will provide initial answers about SMDM quality using a questionnaire. Low response rates to postal questionnaires may limit the available data for this analysis. However, the use of prepaid return envelopes and a reminder should increase the response rate to questionnaires.

## Ethics statement

On 6 December 2021, the protocol received a favorable opinion from the French Committee for the Protection of Persons (2021-A01583-38). The CRCDC will distribute information on the study protocol to healthcare professionals (GPs, gynecologists, midwives) in the region by post 3 months before the intervention. A separate letter informing physicians randomized to the intervention arm of their right not to participate in the research will be sent out during the same period. A letter will be sent informing patients in each arm about their participation in the main study and their opportunity to object. A letter will also be sent inviting patients to answer the ancillary study questionnaires for the and advising them about their freedom of choice to participate. The CRCDC will collect all objections to participation *via* the Data Protection Officer (DPO). Women and GPs will be able to apply to the DPO to be informed of the overall research results.

## Author contributions

Study concept and design: SH, A-SB, EF, and CR. Acquisition, analysis, or interpretation of the data: SH, AG, DT, A-SB, KB, AL, EB, FN, and CR. Drafting of the manuscript: SH, DT, EF, AG, and CR. Critical revision of the manuscript for important intellectual content: SH, EF, and CR. Statistical analysis: AG. Administrative, technical, or material support: A-SB, KB, AL, EB, FN, and CR. Study supervision: SH and CR. The corresponding author attests that all listed authors meet the authorship criteria and that no others meeting this criterion have been omitted. All authors read and approved the final manuscript. The lead author affirms that this manuscript is an honest, accurate, and transparent account of the study being reported; that no important aspects of the study have been omitted; and that any discrepancies from the study as planned (and, if relevant, registered) have been explained. All authors contributed to the article and approved the submitted version.

## Supplementary material

Supplementary Material 1 Objectives, judgement criteria, data collection and source of the data collected

Supplementary Material 2 Characteristics of treatments and examinations carried out collected to assess the woman’s state of health
