# Supplementary material for: Breast cancer: a randomized controlled trial assessing the effect of a decision aid on mammography screening uptake: study protocol
Source: Front Oncol. 2023 Apr 24;13:1128467. doi: 10.3389/fonc.2023.1128467 (PMC10165111; doi:10.3389/fonc.2023.1128467)
Supplement: Supplementary Material 1 — Objectives, judgement criteria, data collection and source of the data collected [file Table_1.docx]

**Supplementary material S1**

**Table S1: Objectives, judgement criteria, data collection and source of the data collected**

| **Objectives** | | **Judgement criteria** | **Data collected** | **Source of the data collected** |
| --- | --- | --- | --- | --- |
| **Principal** | **Assess if:**  the sending of information 1) providing women and general practitioners with an DA, and 2) inviting women to consult their general practitioner for shared decision-making, simultaneously with the sending of the letter of invitation to women's OS (arm « Decision aid for organised cancer screening »), may not decrease the proportion of women who get tested, compared to sending an invitation letter to the women's organization (group « Standard organized cancer screening »). | Proportion of women aged 50-74 who had a screening mammogram 18 months after the procedure. | dates of screening mammography within 24 months before inclusion and within 18 months after inclusion. | Regional organisation in charge of cancer screening in Pays de la Loire (called CRCDC) |
| **Secondary** | **Assess the predictive factors for the use of screening** | Participation rate, 18 months after the intervention, according to age, socio-economic status, medical follow-up, presence of chronic disease, presence of psychopathic disease, previous participation in screening. | Age of the woman, Proxies of socio-economic status (Complementary health insurance status and French DEPrivation index of the place of residence), Number of consultations with various health professionals (GPs, midwives, gynecologists), Number of visits to home by, number of taxi or medical transport journeys, delivery of certain reimbursed treatments and examinations carried out (See Supplementary material S2) over the period of 18 months before and after the intervention,  Number of bilateral screening mammograms performed since the woman became eligible, the time between the last participation in the OS and the start of the intervention, and the number of screenings performed for colon cancer since the woman became eligible. | Health insurance system  Regional organisation in charge of cancer screening in Pays de la Loire (called CRCDC) |
|  | **Measure the implementation of the SMDM and assess the level of decisional conflict within 2 months after the intervention** | Scores obtained on the SDM-Q9 questionnaire and on the DCS scale, responses regarding intention to participate in OS and knowledge of OS, 2 months after the start of the intervention. | Answers to each item,  Percentage of women who reported having consulted the website and having discussed screening with a healthcare professional | Women |

DA: Decision Aid

OS: Organised Screening

SMDM: Shared Medical Decision Making
