# Supplementary material for: Breast cancer: a randomized controlled trial assessing the effect of a decision aid on mammography screening uptake: study protocol
Source: Front Oncol. 2023 Apr 24;13:1128467. doi: 10.3389/fonc.2023.1128467 (PMC10165111; doi:10.3389/fonc.2023.1128467)
Supplement: Supplementary Material 2 — Characteristics of treatments and examinations carried out collected to assess the woman’s state of health [file Table_2.docx]

**Supplementary material S2**

**Table S2: Characteristics of treatments and examinations carried out collected to assess the woman's state of health**

| **Characteristics of the data collected according to the Charlson comorbidity index** | **Response options** |
| --- | --- |
| Delivery of at least 1 antiplatelet treatment | Yes / No |
| Delivery of at least 3 treatments relating to cardiovascular pathology |  |
| Delivery of at least 1 COPD treatment |  |
| Delivery of oxygen therapy at home |  |
| Delivery of at least 1 ulcer or GERD treatment |  |
| Reimbursement of at least 4 αFP assays (0320) AND Reimbursement of at least 2 liver ultrasounds (HLQM001) |  |
| Reimbursement of at least 2 HBA1c assays |  |
| Reimbursement of at least 8 urea + serum creatinine assays (0593) |  |
| Delivery of at least 1 cancer treatment |  |
| Reimbursement of at least 1 tumour marker assay (ACE 7327, SCC 0818, CA19-9 7323) |  |
| Delivery of at least 1 anti-HIV treatment |  |
| Delivery of at least 1 antipsychotic treatment |  |
| Delivery of at least 1 psychiatric treatment |  |
| Delivery of at least 3 different psychiatric treatments |  |
